# Common Genetic Variants of Response to Hepatitis B Vaccines Correlate with Risks of Chronic Infection of Hepatitis B Virus: A Community-Based Case-Control Study

**DOI:** 10.3390/ijms24119741

**Published:** 2023-06-04

**Authors:** Tzu-Wei Wu, Chao-Liang Chou, Chuen-Fei Chen, Li-Yu Wang

**Affiliations:** 1 Department of Medicine, MacKay Medical College, New Taipei City 252, Taiwan; tzuweiwu@mmc.edu.tw (T.-W.W.); chaoliangchou@gmail.com (C.-L.C.); cfchen@mmc.edu.tw (C.-F.C.); 2 Department of Neurology, MacKay Memorial Hospital, New Taipei City 251, Taiwan

**Keywords:** case-control study, community-based study, hepatitis B, hepatitis B vaccination, hepatitis B virus, human leukocyte antigen, single nucleotide polymorphism

## Abstract

Hepatitis B (HB) vaccination effectively reduces the risks of chronic infection with the hepatitis B virus (HBV). It is unknown whether there is a common genetic determinant for response to the HB vaccine and susceptibility to chronic HBV infection. This case-control study, which included 193 chronic HBV carriers and 495 non-carriers, aimed to explore the effects of the most significant single nucleotide polymorphisms (SNPs) in response to the HB vaccine on the risks of chronic HBV infection. Out of 13 tested SNPs, the genotype distributions of four SNPs at the human leukocyte antigen (HLA) class II region, including rs34039593, rs614348, rs7770370, and rs9277535, were significantly different between HBV carriers and non-carriers. The age-sex-adjusted odds ratios (OR) of chronic HBV infection for rs34039593 TG, rs614348 TC, rs7770370 AA, and rs9277535 AA genotypes were 0.51 (95% confidence interval [CI], 0.33–0.79; *p* = 0.0028), 0.49 (95% CI, 0.32–0.75; *p* = 6.5 × 10^−4^), 0.33 (95% CI, 0.18–0.63; *p* = 7.4 × 10^−4^), and 0.31 (95% CI, 0.14–0.70; *p* = 0.0043), respectively. Multivariable analyses showed that rs614348 TC and rs7770370 AA genotypes were significantly independent protectors against chronic HBV infection. The multivariable-adjusted ORs for subjects with none, either one, or both of the protective genotypes were 1.00 (referent), 0.47 (95% CI: 0.32–0.71; *p* = 3.0 × 10^−4^), and 0.16 (95% CI: 0.05–0.54; *p* = 0.0032), respectively. Among eight HBeAg-positive carriers, only one of them carried a protective genotype. This study shows that response to the HB vaccine and susceptibility to chronic HBV infection share common genetic determinants and indicates that HLA class II members are the main responsible host genetic factors.

## 1. Introduction

Chronic hepatitis B (HB) virus (HBV) is common, with a global prevalence of 3.9%, and is more frequent in East Asian populations [1]. It is one of the main causes of deaths and diseases worldwide and is linked to a significant increase in the risk of developing end-stage liver diseases, such as cirrhosis and liver cancer [2,3]. In 2017, approximately 800,000 deaths were caused by HBV, including 90,000 cases of acute hepatitis, 325,400 cases of liver cancer, and 384,000 cases of cirrhosis and other chronic liver diseases [4]. Chronic HBV infection also caused a loss of 28.3 million disability-adjusted life years and 24.4 million years of life in 2017 [5]. Thus, reducing HBV infections in susceptible individuals and reducing disease progression in HBV-infected individuals are crucial for global health.

The implementation of the universal HB vaccination program in Taiwan has led to a decrease in the rates of chronic HBV infection, from over 10% in the 1980s to less than 2% in recent years [6,7]. This has also resulted in a notable decrease in the incidence of liver cancer in children and young adults in Taiwan. As compared with subjects born before the universal HB vaccination program at ages 6–9, 10–14, 15–19, and 20–26 years, the rate ratios of hepatocellular carcinoma for subjects born after the program were 0.26, 0.34, 0.37, and 0.42, respectively [8]. Unfortunately, not many countries have adopted such programs until recently, and only half of the countries and territories have reached the Global Vaccine Action Plan target for HB vaccination coverage [9]. Furthermore, approximately 300 million individuals globally are HBV carriers [1]. As a result, new HBV infections inevitably occur, and an etiologic study on HBV infection seems relevant and urgent.

The universal HB vaccination program in Taiwan provides evidence that HB vaccines effectively induce host immunological responses against chronic HBV infection when contracting HBV and subsequently block the associated pathogenesis. Accordingly, studies on responses to HB vaccines may provide critical clues for the prevention of chronic HBV infection. Recently, several genome-wide association studies (GWAS) have identified a few single nucleotide polymorphisms (SNPs) that are correlated with responses to HB vaccines [10,11,12,13,14]. It is plausible that these genetic markers may also be associated with susceptibility to chronic HBV infection. Considering this, we conducted this community-based genetic association study to explore the relationships between these common genetic variants and the susceptibility to chronic infection in HBV-infected individuals.

## 2. Results

### 2.1. Clinical Characteristics of Study Subjects

**Figure 1:**
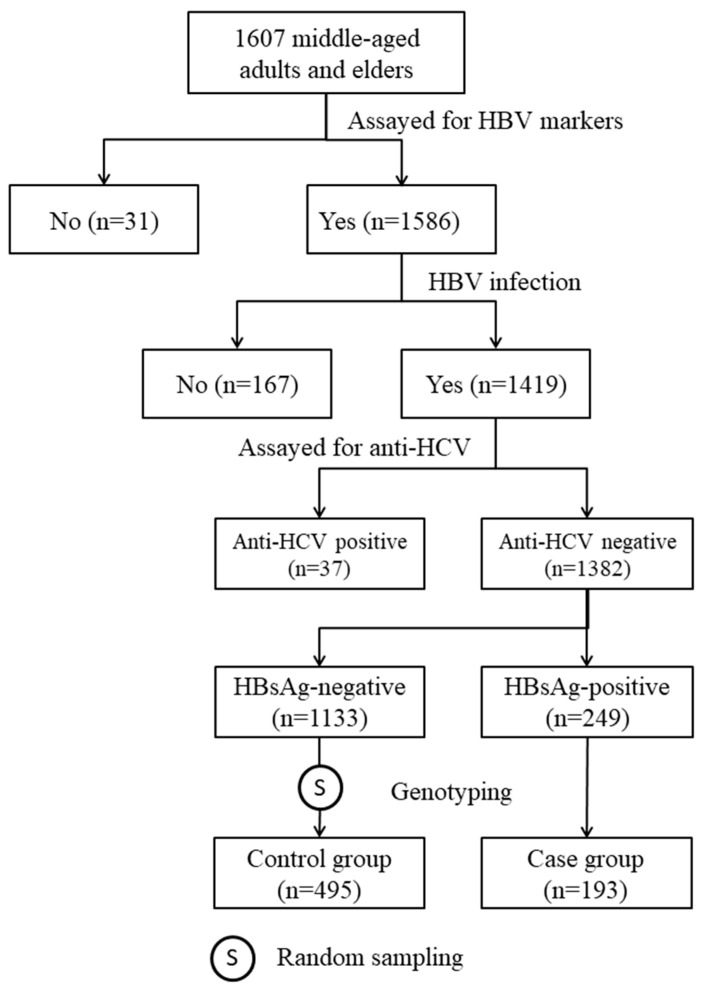
Steps of subject selection.

The subjects of the study were selected from participants in our previous community-based cohort study [15]. In the cohort, there were 1382 anti-hepatitis C virus (anti-HCV)-negative HBV-infected individuals. All HBV carriers with DNA samples available for genotyping (n = 193) and 495 randomly selected non-carriers were included in this case-control study (Figure 1). Among the HBV carriers, 191 (99.0%) of them were also positive for anti-body to HB core antigen (HBcAb; Table 1). The positive rates of HB e antigen (HBeAg) and anti-body to HBeAg (HBeAb) in HBV carriers were 4.3% and 85.3%, respectively. Among the non-carrier controls, the seropositive rates of anti-body to HB surface antigen (HBsAb) and HBcAb were 97.8% and 95.0%, respectively. Table 1 also shows that, as compared with the non-carrier controls, HBV carriers were significantly younger (50.1 ± 8.0 vs. 56.4 ± 9.1 years, *p* < 0.0001) and had a significantly higher mean level of ALT (27.1 ± 17.3 vs. 24.1 ± 14.4 IU/L, *p* = 0.030). There was no significant difference in other baseline clinical and anthropometric measurements between the cases and the controls.

### 2.2. Genotype Distributions and Genotypic Effects of the Tested SNPs

All 13 tested SNPs had a call rate >95%, a minor allele frequency >10.0%, and a *p*-value of the Hardy–Weinberg equilibrium test >0.05 (Table 2). The genotype distributions of rs34039593, rs614348, rs7770370, and rs9277535 were significantly different between the case and control groups. As compared with the non-carrier controls, the relative frequencies of the minor alleles of these four SNPs were significantly lower in the HBV carriers (rs34039593 G: 10.6% vs. 15.9%, *p* = 0.013; rs614348 C: 11.7% vs. 18.6%, *p* = 0.0022; rs7770370 A: 32.1% vs. 40.2%, *p* = 0.0068; rs9277535 A: 26.9% vs. 33.2%, *p* = 0.029). There was no significant difference in the genotype distributions of the remaining nine SNPs between the cases and the controls. Further genotypic effect analyses of the four significant SNPs showed that rs34039593 TG, rs614348 TC, rs7770370 AA, and rs9277535 AA genotypes were associated with significantly decreased risks of chronic HBV infection. The corresponding crude ORs were 0.52 (95% CI: 0.34–0.79; *p* = 0.0023), 0.51 (95% CI: 0.34–0.75; *p* = 7.0 × 10^−4^), 0.38 (95% CI: 0.21–0.67; *p* = 0.0020), and 0.34 (95% CI: 0.16–0.74; *p* = 0.0063), respectively.

### 2.3. Association Analyses with Outcomes of HBV Infection

After controlling for the effects of age and sex, these four genotypes remained correlated with significantly lower risks of chronic HBV infection (Table 3). Further multivariable analyses revealed that the rs614348 TC and rs7770370 AA genotypes showed significantly protective effects against chronic HBV infection. The corresponding adjusted ORs were 0.50 (95% CI: 0.33–0.77; *p* = 0.0016) and 0.35 (95% CI: 0.19–0.68; *p* = 0.0016), respectively.

Table 4 shows that the HBV carriers had a significantly lower mean of the 4-locus genetic protective score (4-GPS) than the non-carrier controls (0.47 ± 0.88 vs. 0.88 ± 1.15, *p* = 8.2 × 10^−7^). As compared with individuals who had no protective genotype, the multivariable-adjusted ORs of chronic HBV infection for individuals who had 1–2 and 3–4 protective genotypes were 0.45 (95% CI: 0.30–0.67; *p* = 1.1 × 10^−5^) and 0.17 (95% CI: 0.05–0.58; *p* = 0.0047), respectively. The multivariable-adjusted OR of chronic HBV infection per 1.0 increase in 4-GPS was 0.65 (95% CI: 0.54–0.79; *p* = 1.1 × 10^−5^). Similar results were observed for the 2-locus GPS (2-GPS). The multivariable-adjusted OR of chronic HBV infection per 1.0 increase in the 2-GPS was 0.45 (95% CI: 0.32–0.63; *p* = 5.6 × 10^−6^).

### 2.4. Genotypes of HBeAg-Positive Carriers

Among HBV carriers, eight (4.3%) of them were also positive for HBeAg. Table 5 shows that only one HBeAg-positive carrier was also positive for HBsAb, and none of them was positive for HBeAb. One HBeAg-positive carrier had an abnormal ALT level, and another two HBeAg-positive carriers had elevated ALT levels close to the upper limit of the normal range. Only one HBeAg-positive carrier (subject no. 629) carried protective genotypes at rs7770370 and rs9277535. The other HBeAg-positive carriers carried no protective genotype at all four polymorphic sites.

## 3. Discussion

In this study, we examined the relationships between the top 13 significant SNPs in response to the HB vaccine and the risks of chronic HBV infection. We found that rs614348 C, rs7770370 A, rs34039593 G, and rs9277356 A alleles were correlated with significantly lower risks of being HBV carriers. Multivariable analysis showed that rs614348 TC and rs7770370 AA genotypes were significantly independent protectors against chronic HBV infection. Additionally, among eight HBeAg-positive carriers, only one of them carried the rs7770370 AA genotype. As far as we know, there is no prior research connecting the polymorphisms of rs614348 and rs34039593 to human health outcomes.

Our previous GWAS showed that human leukocyte antigen-DP (HLA-DP) is the key region for genetic determinants of response to the HB vaccine [10]. We searched the GWAS catalog database [16] and found there were only four associated GWAS studies [11,12,13,14]. These studies reported 26 tagged SNPs, 18 of which had a *p*-value < 10^−6^ and are located in chromosome 6 30.80–33.08 M bp. More importantly, 14 of these SNPs are located in the HLA class II region. Our [10] and previous GWAS [11,12,13,14] findings demonstrated the central roles of HLA class II members in responses to HB vaccines. It is also noted that HLA class II members may also play critical roles in HBV chronic infection, clearance, and responses to anti-viral therapies [17].

In the study, four SNPs were found to be significantly correlated with the risk of chronic HBV infection. The ORs of HBV carriers were significantly lower for the rs7770370 AA and rs9277535 AA genotypes. The directions of effects of the rs7770370 A and rs9277535 A alleles were consistent with both our [10] and a Korean study on the response to the HB vaccine [18]. SNP rs614348 is nearly completely linked with rs477515 in Southern Han Chinese [19]. SNP rs477515 was reported as the most significant SNP for response to the HB vaccine in Chinese Han populations. As compared with the C allele, the T allele was associated with a significantly higher OR (2.05; 95% CI: 1.95–2.41) of non-response to the HB vaccine [12]. Additionally, rs34039593 was reported as one of the lead SNPs for response to a HB vaccine in the Japanese, with the G allele linked to higher anti-HBs levels [13]. Our findings are consistent with previous genetic association studies on response to the HB vaccine.

Previous genetic studies support the significance of rs9277535 and rs7770370 polymorphisms with HBV chronic infection or clearance. Kamatani et al. were the first to report that rs9277535 was the most significantly associated SNP with chronic HBV infection, with the A allele correlated with a significantly lower risk (OR = 0.57; 95% CI: 0.52–0.62) [20]. This finding was consistently replicated in multiple GWAS studies [21,22,23,24]. A meta-analysis including 22,065 chronic HBV infection cases and 23,500 controls from 32 datasets showed that the rs9277535 A allele had a summary OR of 0.60 (95% CI: 0.57–0.63) for chronic HBV infection compared to the G allele [25]. A recent GWAS with 1943 chronic HBV infection cases and 9571 controls identified a cluster of 450 significant SNPs in the HLA class II region [26], among which rs7770370 was the tagged genetic marker and the A allele was correlated with a significantly lower risk of chronic HBV infections [26]. More recently, a community-based study that enrolled more than 0.5 million adults aged 30–79 years found that the rs7770370 A allele and the rs9277535 A allele were both correlated with significantly lower risks of HBsAg positivity [27]. There is no report regarding the health effects of the rs614348 and rs34039593 polymorphisms, except for their relationships with responses to the HB vaccine. 

There are three lines of evidence to support the notion that polymorphisms of rs614348, rs7770370, rs34039593, and rs9277535 may contribute to the susceptibility to chronic HBV infection. First, we used the Ensemble Genome Browser [28] to retrieve the linkage disequilibrium (LD) data of these significant SNPs in the 1000 Human Genome Project Phase 3 Southern Han Chinese [19]. We found that all four of these SNPs are closely linked with nearby functional SNPs (Supplementary Table S1). SNP rs614348 is located in the intergenic region of the *HLA-DRB1* and *-DQA1* genes. It is closely linked with two missense variants (*HLA-DQA1* rs75983419 and rs74379225), one synonymous variant (*HLA-DRB1* rs201125976), and another thirteen functional SNPs. SNP rs7770370 is a non-coding transcript exon variant of the *HLA-DPB1* gene and is also closely linked with another non-coding transcript exon variant of the *HLA-DPB1* gene (rs7770371). SNP rs9277535 is located at the 3′-untranslated region (3′-UTR) of the *HLA-DPB1* gene. It is closely linked with two splice region variants *(HLA-DPB1* rs9277453 and rs9277454), seven missense variants (*HLA-DPB1* rs9277471, rs1042335, rs1042187, rs9277356, rs9277355, rs9277354, and rs1042169), and three synonymous variants (*HLA-DPB1* rs1042212, rs1042331, and rs1071597). Additionally, there are close linkages between the rs9277535 polymorphism and 60 3′-UTR variants of *HLA-DPB1* [19]. SNP rs34039593 is located in the intergenic region of *HLA-DRB1* and *-DQA1* genes and is closely linked with one *HLA-DRB1* missense variant (rs17879995; r^2^ = 0.95) and four regulatory region variants. 

Second, we accessed the expression data in human immune cells [29] using the Ensemble Genome Browser [28] and found that the expression levels of the associated HLA class II genes are significantly influenced by these four significant SNPs. There is a perfect linkage between rs614348 C/T and rs9272545 G/T polymorphisms in Southern Han Chinese. SNP rs9272545 is a 3′-UTR variant of *HLA-DQA1*, and the G allele was correlated with significantly higher expression levels of HLA-DQA1 in multiple B cells, T cells, and monocytes compared to the C allele [29]. Furthermore, expression levels of HLA-DQB1, -DQB1-AS1, -DQB2, and -DRB5 in multiple immune cells were also significantly correlated with the rs9272545 polymorphism ([29]; Supplementary Table S2). The rs7770370 A allele is correlated with significantly higher expression levels of HLA-DPA1 in memory regulatory T cells, naïve B cells, and several monocytes. The rs7770370 A allele also correlates with a higher expression level of HLA-DPB1 in monocytes. Additionally, as compared with the rs7770370 G allele, the expression levels of HLA-DMA, -DMB, -DQA2, -DQB1, -DQB1-AS1, -DQB2, -DRB1, and -DRB5 are also significantly increased for the A allele in multiple B cells, T cells, and monocytes ([29]; Supplementary Table S2). 

The rs9277535 A allele is correlated with higher expression levels of HLA-DPB1 in multiple B cells, T cells, and monocytes. In addition, the expressions of HLA-DMB, -DPA1, -DQA1, -DQB1, and -DQB1-AS1 in many immune cells are also influenced by the rs9277535 polymorphism ([29]; Supplementary Table S2). When using the nearest regulatory region variant rs3129753 as the surrogate for rs34039593 (r^2^ = 0.95), significant correlations with the expression levels of several HLA class II members were observed. For example, as compared with the rs3129753 G allele, the expression levels of *HLA-DRB1* and *-DQA1* were significantly increased in Th1, Th2, Th17, regulatory, CD4+, and CD8+ T cells, B cells, and several monocytes ([29]; Supplementary Table S2). It is therefore reasonable to hypothesize that the rs34039593 polymorphism may influence the expression of many HLA class II genes in immune cells. 

Our previous study showed that both rs777037 A and rs9277535 A alleles are positively correlated with HLA-DPB1*protective alleles, including 02:01, 02:02, 03:01, and 04:01, and are negatively correlated with HLA-DPB1*risk alleles, including 05:01 and 09:01 [10]. Moreover, we also found that the *HLA*-DPA1*02:02:02-DPB1*risk haplotype was positively correlated and the *HLA*-DPA1*01:03:01-DPB1*protective haplotype was negatively correlated with the risks of non-response to the HB vaccine [30]. Recent studies found that the *HLA*-DPA1*01:03-DPB1*04:02 haplotype is correlated with high binding affinities for a large proportion of HBV epitopes [13,26]. On the contrary, the *HLA*-DPA1*02:02-DPB1*05:01 haplotype is correlated with poor binding affinities [13].

This study found no significant correlation between nine candidate SNPs and the risk of chronic HBV infection. One reason could be that except for rs3135363 and rs383006, the other seven SNPs were identified by the same Japanese GWAS study that enrolled subjects who received a HB vaccine designed based on HBV genotype C [13]. However, the most common HBV genotype in Taiwan, with a prevalence of 71%, is genotype B [31]. This suggests that the host genetic factors affecting immune responses to HBV genotypes B and C are likely different. The major antigenic determinants of these genotypes may also vary. Conversely, rs34039593 was identified as a tagged SNP by the same Japanese GWAS study [13] and was found to be related to the risk of chronic HBV infection in this study. It seems that the rs34039593 polymorphism confers immune responses to both HBV genotype B and genotype C. However, blood samples from more than half of the HBV carriers in this study were not enough for HBV genotyping. Further research is needed to test this hypothesis.

In conclusion, we found that four of thirteen candidate SNPs, which showed significant or promising associations with responses to HB vaccines, were significantly correlated with the risks of chronic HBV infection. Notably, the frequencies of the minor alleles of these four significant SNPs were significantly lower in the HBV carriers, and the rs614348 TC and rs7770370 AA genotypes showed significantly protective effects against chronic HBV infection. The results indicate that both the response to HB vaccination and the susceptibility to chronic HBV infection have shared genetic determinants.

## 4. Materials and Methods

### 4.1. Study Subjects 

The subjects of this study were selected from participants in our previous community-based study [15]. From September 2010 to May 2012, a total of 1607 middle-aged adults and elders voluntarily gave informed consent and received physical examinations. At the date of enrollment, a fasting blood sample was drawn from each participant for clinical evaluation and to determine their HBV markers, including HBsAg, HBcAb, HBsAb, and antibody to hepatitis C virus (anti-HCV). Out of the 1607 participants, 1382 individuals who were positive for at least one of the three HBV markers and negative for anti-HCV were eligible for the genetic association study (Figure 1). Among 249 HBsAg-positive subjects, 193 had DNA samples for genotyping and were included as cases. The controls were 495 subjects randomly selected from those who had DNA samples and tested negative for HBsAg but positive for HBcAb and/or HBsAb (Figure 1).

### 4.2. Serologic Testing

In the study, the serostatus of HBsAg and HBcAb was determined using microparticle enzyme immunoassays (MEIA) with commercial kits AxSYN HBsAg (V2) and CORE 2.0 (Abbott Diagnostics, North Chicago, IL, USA), respectively. The titer of HBsAb in the blood samples was quantified by a commercial kit, AxSYN AUSAB (Abbott Diagnostics), with a detection limit of 1.0 mIU/mL. HBeAg, HBeAb, and anti-HCV were assayed by chemiluminescent microparticle immunoassay (CMIA) with commercial kits ARCHITECT HBeAg, Anti-HBe, and Anti-HCV (Abbott Core Laboratories, North Chicago, Illinois A, USA), respectively. 

In the study, all subjects were born before 1972 and were 12 or older before the implementation of the universal HB vaccination program in Taiwan. It can be assumed that none of them received the HB vaccination. Accordingly, HBV infection was defined as a positive result for any of the three HBV markers (HBsAg, HBsAb, and HBcAb). HBV chronic carriers were defined as subjects who were seropositive for HBsAg, regardless of the serostatus of HBsAb or HBcAb. HBV non-carriers were defined as subjects who were seropositive for either HBsAb or HBcAb but negative for HBsAg.

### 4.3. SNP Selections

We searched the GWAS catalog [16] and identified 4 GWAS on responses to HB vaccines [11,12,13,14]. These studies reported 18 SNPs with a *p*-value less than 10^−6^, including rs12527394, rs9267665, rs3132969, rs9268202, rs4248166, rs3135363, rs2395179, rs9268831, rs477515, rs34039593, rs9273062, rs7745040, rs1015166, rs9277176, rs9277356, rs9277464, rs9277535, and rs9277549. Additionally, our previous GWAS study identified 3 SNPs (rs35953215, rs3830066, and rs7770370) that showed promising associations with responses to HB vaccines [10].

We used the Ensemble Genome Browser [29] to retrieve the linkage disequilibrium (LD) data of these candidate SNPs in the 1000 Human Genome Project Phase 3 Southern Han Chinese [19]. The LD data show that there are close linkages between rs3132969 and rs9268202 (r^2^ = 0.83), between rs4248166 and rs2395179 (r^2^ = 0.86), and among rs9277356, rs9277464, rs9277535, and rs9277549 (all pairwise r^2^ > 0.94). Our previous GWAS study also showed that SNP rs35953215 is completely linked, with an r^2^ of 1.00, with rs3830066 [10]. To reduce the influence of multiple comparisons, we used the SNP with the lowest *p*-value in each LD block as the candidate SNP, resulting in 15 independent SNPs (rs12527394, rs9267665, rs9268202, rs3135363, rs2395179, rs9268831, rs477515, rs34039593, rs9273062, rs7745040, rs1015166, rs9277176, rs3830066, rs9277356, and rs7770370) eligible for the genetic association study.

SNPs rs3135363, rs9268831, rs34039593, rs1015166, rs3830066, and rs7770370 were the designed SNPs of the Axiom CHB1 plate (Thermo Fisher Scientific, Waltham, MA, USA). For the other SNPs, we used their highest and nearest LD SNPs as surrogates. The surrogate SNPs for rs12527394, rs9268202, rs2395179, rs477515, rs7745040, rs9277176, and rs9277356 were rs4947302, rs9268176, rs3129846, rs614348, rs6457620, rs3097662, and 9277535, respectively. SNPs rs9267665 and rs9273062 were excluded due to the lack of a suitable surrogate. As a result, 13 SNPs were eligible for the genetic association study. Information about these SNPs and the characteristics of the typed SNPs are shown in Table 6.

### 4.4. Genotyping 

The genomic DNA of each subject was extracted from EDTA-containing whole blood samples using a semi-automated extraction system, Smart LabAssist (Taiwan Advanced Nanotech Inc., Tau-Yuan County, Taiwan), with a TANBead Blood DNA plate (Taiwan Advanced Nanotech Inc.). The genotyping of all samples was performed at the National Center for Genomic Medicine, Academia Sinica, Taiwan, using the Axiom CHB1 array plate.

The genotype information for the 13 tested SNPs in HBV carriers and non-carriers is displayed in Table 2. The eligibility of SNPs for association analyses was a call rate >95%, a *p*-value of the Hardy–Weinberg equilibrium (HWE) test in the controls >0.001, and a minor allele frequency >3%. 

### 4.5. Statistical Analyses

We used Pearson’s chi-square test to assess whether there was a significant difference in the genotype distributions of the candidate SNPs between HBV carriers and non-carrier controls. All significant SNPs were subject to analysis for their genotypic effects by four models, including major allele dominant, minor allele dominant, co-dominant, and additive. The most significant genotypic effect model of each significant SNP was subject to multivariable analyses. We used unconditional logistic regression to calculate crude and adjusted odds ratios (OR) and their 95% confidence intervals (CI) of chronic HBV infection for specific alleles or genotypes. The significance level of this study was set at 0.05 divided by the number of candidate SNPs. We further generated multi-locus genetic protective scores (GPSs) by summing the number of protective alleles or genotypes for each individual and then assessed their associations with chronic HBV infection. All statistical analyses were performed using SAS 9.4 (SAS Institute Inc., Cary, NC, USA).

## Figures and Tables

**Table 1:** Baseline clinical characteristics of HBV carriers and non-carrier controls.

	HBV Carriers (n = 193)		Non-Carrier Controls (n = 495)		*p*-Value
HBV markers	n	(%)	n	(%)	
HBsAg+	193	(100.0)	0	(0.0)	-
HBsAb+	20	(10.4)	484	(97.8)	<0.0001
HBcAb+	191	(99.0)	470	(95.0)	0.015
HBeAg+	8	(4.3)	ND		-
HBeAb+	163	(85.3)	ND		-
Male sex	93	(48.2)	247	(49.9)	0.69
Cigarette smoking	39	(20.2)	83	(16.7)	0.39
Alcohol drinking	18	(9.3)	33	(6.6)	0.30
Schooling >12 years	67	(34.7)	138	(28.0)	0.085
	Mean	(SD)	Mean	SD	
Age (years)	50.1	(8.0)	56.4	(9.1)	<0.0001
AST (IU/L)	26.4	(10.1)	24.9	(12.9)	0.098
ALT (IU/L)	27.1	(17.3)	24.1	(14.4)	0.030

Notes: -, not calculated; ALT = alanine aminotransferase; AST = aspartate aminotransferase; HBcAb = anti-body to hepatitis B core antigen; HBeAb = anti-body to hepatitis B e antigen; HBeAg = hepatitis B e antigen; HBsAb = anti-body to hepatitis B surface antigen; HBsAg = hepatitis B surface antigen; HBV = hepatitis B virus; ND = not determined; SD = standard deviation.

**Table 2:** Genotype distributions of 13 tested SNPs between HBV carriers and non-carrier controls.

Typed SNP	Position (GRCh38)	Candidate SNP	Allele A/B ^1^	HBV Carriers (n = 193)		Non-Carrier Controls (n = 495)		*p*-Value ^2^
				A (%)	AA/AB/BB	A (%)	AA/AB/BB	
rs4947302	6:31106351	rs12527394	T/C	23.1	4/81/108	21.9	23/170/302	0.076
rs9268176	6:32306302	rs9268202	T/C	17.9	9/51/133	20.5	18/166/311	0.18
rs3135363	6:32421871	rs3135363	G/A	39.6	33/90/70	38.6	72/237/185	0.71
rs3129846	6:32428698	rs2395179	G/A	21.0	7/67/119	21.1	20/168/307	0.96
rs9268831	6:32459971	rs9268831	C/T	40.7	30/97/66	36.3	57/244/195	0.27
rs34039593	6:32602534	rs34039593	G/T	10.6	4/33/156	15.9	8/141/346	0.0084
rs614348	6:32606100	rs477515	C/T	11.7	4/37/152	18.6	12/159/322	0.0025
rs6457620	6:32696222	rs7745040	G/C	39.1	31/90/72	39.6	83/224/186	0.95
rs1015166	6:32830954	rs1015166	T/C	30.1	19/78/96	27.2	33/202/260	0.36
rs3097662	6:33053000	rs9277176	C/T	11.9	2/42/149	13.7	8/119/368	0.68
rs3830066	6:33069410	rs3830066	C/G	21.8	9/66/118	27.9	35/205/254	0.062
rs7770370	6:33081144	rs7770370	A/G	32.1	13/98/82	40.2	79/238/178	0.0050
rs9277535	6:33087084	rs9277356	A/G	26.9	8/88/97	33.2	55/217/223	0.016

^1^ A, minor allele; B, major allele. ^2^ *p*-value of the chi-square test for genotype distributions between case and control groups. Notes: CI = confidence interval; HBV = hepatitis B virus; OR = odds ratio; SNP = single nucleotide polymorphism.

**Table 3:** Association analyses with risks of chronic HBV infection.

	Age-Sex-Adjusted		Multivariable	
Protective Genotype	OR	(95% CI)	OR ^1^	(95% CI)
rs34039593 TG	0.51 **	(0.33–0.79)	-	
rs614348 TC	0.49 **	(0.32–0.75)	0.50 **	(0.33–0.77)
rs7770370 AA	0.33 **	(0.18–0.63)	0.35 **	(0.19–0.68)
rs9277535 AA	0.32 **	(0.14–0.70)	-	

^1^ ORs were adjusted for age and sex. Notes: -, not included; CI = confidence interval; HBV = hepatitis B virus; OR = odds ratio; **, 0.0001 < *p* < 0.005.

**Table 4:** Association analyses for chronic HBV infection with multi-locus genetic protective scores (GPS).

	HBV Carriers (n = 193)		Non-Carrier Controls (n = 495)		Multivariable	
Variable	n	(%)	n	(%)	OR ^1^	(95% CI)
4-locus GPS ^2^						
Mean (SD)	0.47	(0.88)	0.88	(1.15)		
GPS						
0	146	(75.7)	284	(57.4)	1.00	
1–2	44	(22.8)	179	(36.2)	0.45 ***	(0.30–0.67)
3–4	3	(1.6)	32	(6.5)	0.17 **	(0.05–0.58)
Per 1.0 GPS					0.65 ***	(0.54–0.79)
2-locus GPS ^3^						
Mean (SD)	0.26	(0.47)	0.48	(0.62)		
GPS						
0	146	(75.7)	289	(58.4)	1.00	
1	44	(22.8)	172	(34.8)	0.47 **	(0.32–0.71)
2	3	(1.6)	34	(6.9)	0.16 **	(0.05–0.54)
Per 1.0 GPS					0.45 ***	(0.32–0.63)

^1^ ORs were adjusted for age and sex. ^2^ Includes rs34039593, rs614348, rs7770370, and rs9277535. ^3^ Includes rs614348 and rs7770370. Notes: CI = confidence interval; HBV = hepatitis B virus; OR = odds ratio; SD = standard deviation; **, 0.0001 < *p* < 0.005; ***, *p* < 0.0001.

**Table 5:** Genotypes and HBV markers of HBeAg-positive carriers.

No.	Age	Sex	HBsAb	HBcAb	HBeAb	AST	ALT	rs34039593	rs614348	rs7770370	rs9277535
112	44.3	M	-	+	-	27	39	TT	TT	AG	AG
255	69.6	F	+	+	-	36	39	TT	TT	AG	AG
261	69.1	F	-	+	-	31	23	TT	TT	GG	GG
398	67.6	M	-	+	-	50	46	TT	TT	AG	AG
418	53.7	F	-	+	-	23	22	TT	TT	GG	GG
569	42.8	F	-	+	-	23	27	TT	TT	AG	AG
626	45.2	F	-	+	-	18	19	TT	TT	GG	GG
629	46.1	F	-	+	-	19	17	TT	TT	AA	AA

**Table 6:** Basic characteristics of candidate and tested SNPs.

Candidate SNP	Position (GRCh38)	Tested SNP	Position (GRCh38)	LD (r^2^)	Type of Variant	Mapped Gene
rs12527394	6:31087241	rs4947302	6:31106351	0.97	intergenic	*C6orf15, RNU6-1133P*
rs9268202	6:32311563	rs9268176	6:32306302	1.00	intronic	*TSBP1, TSBP1-AS1*
rs3135363	6:32421871	rs3135363	6:32421871	1.00	intergenic	*BTNL2, TSBP1-AS1, HLA-DRA*
rs2395179	6:32439525	rs3129846	6:32428698	0.97	intergenic	*BTNL2, TSBP1-AS1, HLA-DRA*
rs9268831	6:32459971	rs9268831	6:32459971	1.00	NCTEV	*HLA-DRB9*
rs477515	6:32601914	rs614348	6:32606100	0.96	intergenic	*HLA-DQA1, HLA-DRB1*
rs34039593	6:32602534	rs34039593	6:32602534	1.00	intergenic	*HLA-DQA1, HLA-DRB1*
rs7745040	6:32696555	rs6457620	6:32696222	0.93	intergenic	*HLA-DQB1, MTCO3P1*
rs1015166	6:32830954	rs1015166	6:32830954	1.00	intronic	*TAP2*
rs9277176	6:33055373	rs3097662	6:33053000	0.97	regulatory	*HLA-DPA1*
rs3830066	6:33069410	rs3830066	6:33069410	1.00	NCTEV	*HLA-DPA1*
rs9277356	6:33080917	rs9277535	6:33087084	0.94	3′-UTR	*HLA-DPB1*
rs7770370	6:33081144	rs7770370	6:33081144	1.00	NCTEV	*HLA-DPA1, HLA-DPB1*

Note: NCTEV = noncoding transcript exon variant; UTR = un-translated region.

## Data Availability

The datasets used and/or analyzed in the current study are available from the corresponding author (Li-Yu Wang) on reasonable request.

## Supplementary Materials

The following supporting information can be downloaded at: https://www.mdpi.com/article/10.3390/ijms24119741/s1.
