# Supplementary material for: Common Genetic Variants of Response to Hepatitis B Vaccines Correlate with Risks of Chronic Infection of Hepatitis B Virus: A Community-Based Case-Control Study
Source: Int J Mol Sci. 2023 Jun 4;24(11):9741. doi: 10.3390/ijms24119741 (PMC10253545; doi:10.3390/ijms24119741)
Supplement: Supplementary file 1 [file ijms-24-09741-s001.zip › ijms-2413161-supplementary.pdf]

Supplementary Table S1. LD SNPs of 4 significant SNPs in southern Han Chinese.

SNP (Type)[1]	LD SNP (Type)[1]
rs34039593 (H)	rs17879995 (B); rs3129753 (G); rs510205 (G); rs6931277 (G); rs6941395 (G).
rs614348 (H)	rs75983419 (B); rs74379225 (B); rs1049068 (C); rs201125976 (C); rs34043628 (D); rs9271554 (D); rs192165457 (E); rs28383359 (E); rs28383364 (E); rs63749607 (E); rs76867035 (E); rs78702533 (E); rs79298326 (E); rs79772777 (E); rs80217639 (E); rs9272545 (E); rs9272562 (E); rs62404117 (F); rs62404119 (F); rs9273165 (F); rs113177970 (G); rs113468765 (G); rs113676242 (G); rs17613367 (G); rs200755517 (G); rs2647079 (G); rs28359885 (G); rs28442287 (G); rs28584179 (G); rs28886951 (G); rs34046799 (G); rs34386495 (G); rs35025478 (G); rs35700251 (G); rs35786457 (G); rs35932914 (G); rs35947801 (G); rs62406302 (G); rs73732214 (G).
rs7770370 (F)	rs7770501 (F).
rs9277535 (E)	rs9277397 (A); rs9277398 (A); rs9277452 (A); rs9277453 (A); rs9277454 (A); rs9277492 (A); rs1042169 (B); rs1042187 (B); rs1042335 (B); rs9277354 (B); rs9277355 (B); rs9277356 (B); rs9277471 (B); rs1042212 (C); rs1042331 (C); rs1071597 (C); rs1042467 (E); rs1042488 (E); rs1042497 (E); rs1042502 (E); rs1042507 (E); rs1042508 (E); rs1042511 (E); rs1042516 (E); rs1042544 (E); rs3091281 (E); rs3091282 (E); rs3097650 (E); rs3117228 (E); rs3117229 (E); rs3128963 (E); rs3128964 (E); rs3128967 (E); rs3128968 (E); rs3130186 (E); rs3130187 (E); rs3130188 (E); rs5875436 (E); rs9277529~rs9277551 (E); rs9277553 (E); rs9277554 (E); rs9277557~s9277559 (E); rs9277562~s9277564 (E); rs9277566 (E); rs9277568 (E); rs928 (E); rs9280317 (E); rs929 (E); rs930~s933 (E); rs935 (E); rs2295118 (F); rs531806432 (F); rs542443316 (F); rs562893885 (F); rs563606896 (F); rs9277399~rs9277414 (F); rs9277417~s9277419 (F).

[1] Type of variant: A, splice polypyrimidine tract variant; B, missense variant; C, synonymous variant; D, TF binding site; E, 3 prime UTR variant; F, non coding transcript exon variant; G, regulatory region variant; H, intergenic variant.

Supplementary Table S2. Correlations of 6 SNPs with significant effects on the expressions of HLA class members in different tissues [26].

SNP	Gene_name	Tissue/cell[1]
rs3129753 (LD with rs34039593)	HLA-DQB1	B-cell_naive, CD4_T-cell_naive, CD8_T-cell_naive, LCL, T-cell, Treg_naive, iPSC, macrophage_Listeria, monocyte_CD16_naive, monocyte_Pam3CSK4, neutrophil
	HLA-DRB1	B-cell_naive, CD4_T-cell_anti-CD3-CD28, CD4_T-cell_naive, CD8_T-cell_naive, LCL, NK-cell_naive, T-cell, Tfh_memory, Th1-17_memory, Th17_memory, Th1_memory, Th2_memory, Treg_memory, Treg_naive, iPSC, macrophage_IFNg, macrophage_Listeria, monocyte, monocyte_CD16_naive, monocyte_IAV, monocyte_LPS, monocyte_Pam3CSK4, monocyte_R848, monocyte_naive, neutrophil
	HLA-DQA1	B-cell_naive, CD4_T-cell_anti-CD3-CD28, CD4_T-cell_naive, CD8_T-cell_anti-CD3-CD28, CD8_T-cell_naive, LCL, macrophage_Listeria, macrophage_Salmonella, monocyte_LPS, monocyte_Pam3CSK4
	HLA-DRB5	B-cell_naive, T-cell, iPSC, monocyte
	HLA-DMA	iPSC
	HLA-DRA	iPSC, monocyte_LPS, monocyte_Pam3CSK4, monocyte_R848
	HLA-DQB1-AS1	B-cell_naive, CD4_T-cell_naive, LCL, NK-cell_naive, T-cell, Th1-17_memory, Th2_memory, Treg_memory, macrophage_Listeria, macrophage_Salmonella, macrophage_naive, monocyte_CD16_naive, monocyte_IAV, monocyte_LPS, monocyte_Pam3CSK4, monocyte_R848, monocyte_naive
	HLA-DQB2	CD4_T-cell_anti-CD3-CD28, CD8_T-cell_anti-CD3-CD28, LCL, Th17_memory, Th2_memory, Treg_memory, macrophage_IFNg, macrophage_Listeria, macrophage_Salmonella, macrophage_naive, monocyte, monocyte_IAV, monocyte_LPS, monocyte_Pam3CSK4, monocyte_R848, monocyte_naive
	HLA-DQA2	B-cell_naive, CD4_T-cell_anti-CD3-CD28, CD4_T-cell_naive, CD8_T-cell_anti-CD3-CD28, CD8_T-cell_naive, LCL, NK-cell_naive, T-cell, Tfh_memory, Th1-17_memory, Th17_memory, Th1_memory, Th2_memory, Treg_memory, Treg_naive, macrophage_IFNg, macrophage_Listeria, monocyte, monocyte_CD16_naive, monocyte_IAV, monocyte_LPS, monocyte_Pam3CSK4, monocyte_R848, monocyte_naive
	HLA-DOB	CD4_T-cell_naive, CD8_T-cell_naive, Liver, NK-cell_naive, T-cell, Th1-17_memory, Th1_memory, Th2_memory, Treg_naive, iPSC, macrophage_IFNg, macrophage_Listeria, macrophage_naive, monocyte, monocyte_Pam3CSK4, neutrophil
rs9272545 (LD with rs614348)	HLA-DMA	iPSC
	HLA-DQA1	B-cell_naive, CD8_T-cell_anti-CD3-CD28, LCL, macrophage_IFNg, macrophage_Listeria, macrophage_naive, monocyte_CD16_naive, monocyte_IAV, monocyte_LPS, monocyte_naive, monocyte_Pam3CSK4, monocyte_R848, T-cell, Th17_memory, Treg_memory, Treg_naive
	HLA-DQA2	macrophage_IFNg
	HLA-DQB1	B-cell_naive, CD4_T-cell_anti-CD3-CD28, CD4_T-cell_naive, CD8_T-cell_anti-CD3-CD28, CD8_T-cell_naive, iPSC, LCL, macrophage_IFNg, macrophage_Listeria, macrophage_naive, macrophage_Salmonella, monocyte_CD16_naive, monocyte_IAV, monocyte_LPS, monocyte_naive, monocyte_Pam3CSK4, monocyte_R848, NK-cell_naive, T-cell, Tfh_memory, Th1-17_memory, Th17_memory, Th2_memory, Treg_memory, Treg_naive

	HLA-DQB1-AS1	B-cell_naive, CD4_T-cell_anti-CD3-CD28, CD4_T-cell_naive, CD8_T-cell_anti-CD3-CD28, LCL, macrophage_Listeria, macrophage_naive, macrophage_Salmonella, monocyte_CD16_naive, monocyte_IAV, monocyte_LPS, monocyte_naive, monocyte_Pam3CSK4, NK-cell_naive, T-cell, Th1-17_memory, Th17_memory, Th2_memory, Treg_memory
	HLA-DQB2	B-cell_naive, macrophage_IFNg, macrophage_Salmonella, monocyte_IAV, monocyte_LPS, monocyte_naive, monocyte_Pam3CSK4, monocyte_R848, NK-cell_naive
	HLA-DRA	iPSC, monocyte_naive
	HLA-DRB5	CD8_T-cell_naive, iPSC, LCL, macrophage_IFNg, macrophage_naive, macrophage_Salmonella, monocyte_IAV, monocyte_LPS, monocyte_naive, monocyte_Pam3CSK4, monocyte_R848, NK-cell_naive, Tfh_memory, Th1_memory, Th2_memory, Treg_memory
rs9277535	HLA-DMB	Th1_memory
	HLA-DPA1	iPSC, LCL, monocyte
	HLA-DPB1	B-cell_naive, CD8_T-cell_naive, iPSC, LCL, macrophage_naive, monocyte, monocyte_CD16_naive, monocyte_IAV, monocyte_LPS, monocyte_naive, monocyte_Pam3CSK4, monocyte_R848, T-cell, Treg_memory
	HLA-DQA1	monocyte_IAV, monocyte_naive, NK-cell_naive
	HLA-DQB1	monocyte_naive
	HLA-DQB1-AS1	monocyte_IAV, monocyte_naive, monocyte_Pam3CSK4, monocyte_R848
rs7770370	HLA-DMA	iPSC, neutrophil, NK-cell_naive
	HLA-DMB	Th1-17_memory
	HLA-DPA1	B-cell_naive, iPSC, LCL, monocyte, monocyte_CD16_naive, monocyte_IAV, monocyte_LPS, monocyte_naive, monocyte_Pam3CSK4, monocyte_R848, Treg_memory
	HLA-DPB1	iPSC, monocyte
	HLA-DQA2	monocyte_IAV, monocyte_LPS, monocyte_naive, monocyte_Pam3CSK4, monocyte_R848
	HLA-DQB1	iPSC, monocyte_LPS, monocyte_naive, monocyte_Pam3CSK4
	HLA-DQB1-AS1	monocyte_IAV, monocyte_LPS
	HLA-DQB2	monocyte_LPS
	HLA-DRB1	iPSC
	HLA-DRB5	iPSC, monocyte

[1] Only those cells with a p-value <0.01 are listed.
